# Dataset for proteomic analysis of *Chlorella sorokiniana* cells under cadmium stress

**DOI:** 10.1016/j.dib.2020.106544

**Published:** 2020-11-18

**Authors:** Antonio León-Vaz, Luis C. Romero, Cecilia Gotor, Rosa León, Javier Vigara

**Affiliations:** a Laboratory of Biochemistry. Faculty of Experimental Sciences. Marine International Campus of Excellence and REMSMA. University of Huelva, 210071 Huelva, Spain; b Instituto de Bioquímica Vegetal y Fotosíntesis. Consejo Superior de Investigaciones Científicas and Universidad de Sevilla. Avenida Américo Vespucio, 49. 41092 Seville. Spain

**Keywords:** Cadmium, Chlorella sorokiniana, Proteomics, Heavy metal

## Abstract

Cadmium is one of the most hazardous heavy metal for aquatic environments and one of the most toxic contaminants for phytoplankton. This work provides the dataset associated with the research publication “Effect of cadmium in the microalga *Chlorella sorokiniana*: a proteomic study” [1]. This dataset describes a proteomic approach, based on the sequential window acquisition of all theoretical fragment ion spectra mass spectrometry (SWATH-MS), derived from exposure of *Chlorella sorokiniana* to 250 µM Cd^2+^ for 40 h, showing the proteins that are up- or downregulated. The processing of data included the identification of the *Chlamydomonas reinhardtii* protein sequences equivalent to the corresponding of *Chlorella sorokiniana* sequences obtained, which made possible to use KEGG Database. MS and MS/MS information, and quantitative data were deposited PRIDE public repository under accession number PXD015932.

## Specifications Table

**Table:** 

Subject area	Biochemistry
Specific subject area	Proteomics, microalgae
Type of data	Figure and table
How data were acquired	Nano-LC MS/MS using Triple TOF® 5600+ System (Sciex, CA, USA). SWATH-MS analysis using Protein Pilot software (version 5.0.1, Sciex) Protein equivalences using Uniprot repository.
Data format	Raw and processed data.
Parameters for data collection	Presence of Cd^2+^, SWATH analysis and equivalences between *Chlorella sorokiniana* and *Chlamydomonas reinhardtii* proteins
Description of data collection	Cultures of *Chlorella sorokiniana* without (Control) and with Cd (250 µM). Extraction of proteins using TRIzol treatment, and SWATH-MS analysis. Identification of equivalent proteins in *Chlamydomonas reinhardtii* by KEGG Database
Data source location	University of Huelva, Huelva (Spain)
Data accessibility	Raw data available at PRIDE repository with the accession PXD015932 https://www.ebi.ac.uk/pride/archive/projects/PXD015932 Processed datasets are in the article and at Mendeley Data repository with the DOI 10.17632/nksvw4ms57.1
Related research article	León-Vaz, A., Romero, L.C., Gotor, C., León, R., Vigara, J., 2021. Effect of cadmium in the microalga *Chlorella sorokiniana*: A proteomic study. Ecotoxicology and Environmental Safety 207, 111301. Doi: 10.1016/j.ecoenv.2020.111301

## Value of the Data

• These data support the information for the modifications observed in the proteomic profile of *C. sorokiniana,* exposed to Cd stress, provided in [1]. This is one of the first proteome studies performed with *C. sorokiniana*, so these data could open a new field of research related to this microorganism.
• These data can be useful for other researchers in order to facilitate their investigation due to the fact that *C. sorokiniana* protein sequences are no available in databases, such as KEGG or PANTHER. Thus, this data could be useful for the quick protein classification and analysis of this microalga proteome.
• The data can be used in order to perform subsequent proteomic studies in *C. sorokiniana* trough the protein equivalences with the model microalga *Chlamydomonas reinhardtii*.

## 1 Data Description

**Fig. 1:**
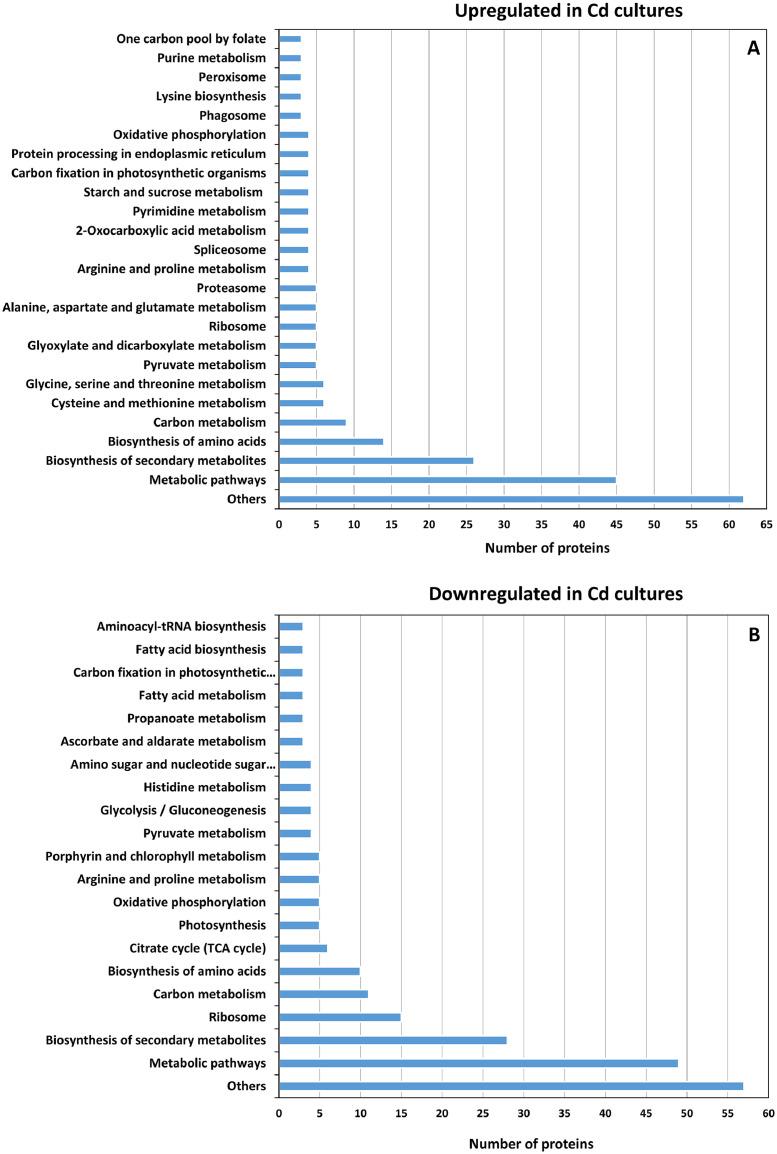
Total of proteins up- (A) and downregulated (B) in Cd-treated *C. sorokiniana* cultures obtained from KEGG database using the equivalent *C. reinhardtii* proteins.

The data presented in this paper show the differential protein expression between three control cultures of *Chlorella sorokiniana* (*C. sorokiniana*), cultivated in standard conditions, and three cultures grown in the presence of 250 µM of Cd. The dataset obtained from the SWATH-MS analysis includes 218 proteins, more abundant in untreated cultures and 255 ones, more abundant in Cd-treated cultures (*p*value < 0.05). The sequence of these *C. sorokiniana* proteins did not appear in protein databases, such as KEGG and PANTHER. Thus, equivalent proteins of the model green microalga *Chlamydomonas reinhardtii* (*C. reinhardtii*) have been used in order to identify the affected proteins in *C. sorokiniana*. The sequences of the genes encoding the upregulated and downregulated proteins in Cd cultures were introduced in KEGG database, and the affected metabolic pathways are presented in Fig. 1. In addition, the equivalence between proteins from *C. sorokiniana* and *C. reinhardtii* is shown in the repository Mendeley Data with the DOI 10.17632/nksvw4ms57.1. The raw data were submitted to ProteomeXChange database with the accession PXD015932.

## 2 Experimental Design, Materials and Methods

### 2.1 Algal strain and culture conditions and crude extract preparation

The *C. sorokiniana* 211–32 strain from the culture collection of the Institute of Plant Biochemistry and Photosynthesis (IBVF; Seville, Spain) was grown mixotrophically in liquid Tris-Acetate-Phosphate (TAP) medium [2], optimized as previously described [3] for this microalga, containing (g L^−1^): 0.006 H_2_KPO_4_, 0.115 HK_2_PO_4_, 0.1 MgSO_4_•7H_2_O, 0.05 CaCl_2_•2H_2_O, 2.49 Tris Base, 1.50 NH_4_Cl, 5.94 mL L^−1^ of acetic acid and 5 mL L^−1^ of traces solution containing (g L^−1^): 10.0 EDTA, 2.28 H_3_BO_3_, 4.40 ZnSO_4_•7H_2_O, 1.02 MnCl_2_•4H_2_O, 1.00 FeSO_4_•7H_2_O, 0.32 CoCl_2_•6H_2_O, 0.32 CuSO_4_•5H_2_O and 0.22 Mo_7_O_24_(NH_4_)_6_•4H_2_O. The addition of cadmium, as CdCl_2_, was done before sterilization to a concentration of 250 μM.

### 2.2 Protein extraction

*C. sorokiniana* cells were harvested by centrifugation at the middle of the exponential phase of growth (40 h), washed and disrupted by sonication. The supernatant obtained was used as protein source and, proteins were precipitated using the TRIzol method [4]. In this method 1 mL of Trizol was added to about 700 µL of crude extract with a concentration of 1.5 mg mL^−1^. The mixture was homogenized during 15 s and incubated at 4 °C for 5 min. After that, 200 µL of chloroform were added for proteins separation, and the mixture was homogenized for 15 s, incubated for 5 min at 4 °C and centrifuged at 14000 x g during 15 min. The upper phase was discarded and 300 µL of pure ethanol were added to organic phase. The solution was agitated and centrifuged at 3000 x g during 10 min. The supernatant was selected and 1 mL of isopropanol was added to it. This solution was incubated 10 min at 25 °C for proteins precipitation. After that time, the mixture was centrifuged at 14000 x g during 15 min and the supernatant was discarded. Proteins pellet was washed 3 times with a 0.3 M guanidine solution in ethanol (95%, v/v), and centrifuged (14000 x g, 5 min, 4 °C). The precipitated obtained was washed with ethanol 90% and resuspended in a 50 mM ammonium bicarbonate: 50% trifluoroethanol, 10 mM DTT, for subsequent SWATH-MS analysis.

### 2.3 Protein relative quantitation by SWATH-MS acquisition and analysis

Protein samples from *C. sorokiniana* 211-32 were alkylated, trypsin-digested, and differences in protein level between control conditions and Cd treated cell a Label Free Quantitative analysis was attended by SWATH-MS methods as previously described [5,6].

The peptide and protein identifications were set to a false discovery rate (FDR) below 0.01 for both peptides and proteins. Finally, peptides with a confidence score above 99% were included in the spectral library.

For relative quantitation using SWATH analysis, the same samples used to generate the spectral library were analysed using a data-independent acquisition (DIA) method. The method consisted of repeating an acquisition cycle of 34 TOF MS/MS scans (230 to 1500 m/z, 100 ms acquisition time) of overlapping sequential precursor isolation windows of 25 m/z width (1 m/z overlap) covering the 400 to 1250 m/z mass range with a previous TOF MS scan (400 to 1250 m/z, 50 ms acquisition time) for each cycle. The extracted ion chromatograms were then generated for each selected fragment ion and the peak areas for the peptides obtained by summing the peak areas from the corresponding fragment ions. Only peptides with an FDR below 5% were used for protein quantitation. Protein quantitation was calculated by adding the peak areas of the corresponding peptides. MarkerView (version 1.2.1, SCIEX) was used for signal normalization in order to test for differential protein abundance between the two groups.

Equivalent proteins of the model green microalga *Chlamydomonas reinhardtii* were obtained using UniProt database. For this purpose, *Chlorella sorokininiana* proteins obtained by SWATH-MS were located in Uniprot database. After that, the sequence was selected and aligned using UniProt BLAST. The equivalent proteins for the model microalga *Chlamydomonas reinhardtii* were selected, after corroborate they had the same function in *Chlorella sorokiniana*.

### 2.4 Mass spectrometry dataset deposit

The mass spectrometry proteomics data have been deposited in the ProteomeXchange Consortium via the PRIDE [7] partner repository with identifier PXD015932.

## CRediT Author Statement

Antonio León-Vaz: conceptualization, investigation and methodology; Luis C. Romero: conceptualization, methodology and writing-review; Cecilia Gotor: review; Rosa León: writing-review and funding acquisition; Javier Vigara: conceptualization; writing-review and editing.

## Declaration of Competing Interest

The authors declare that they have no known competing financial interests or personal relationships that could have appeared to influence the work reported in this paper.
